# Cefiderocol-Based versus Colistin-Based Regimens for Severe Carbapenem-Resistant *Acinetobacter baumannii* Infections: A Propensity Score-Weighted, Retrospective Cohort Study during the First Two Years of the COVID-19 Pandemic

**DOI:** 10.3390/microorganisms11040984

**Published:** 2023-04-10

**Authors:** Maria Mazzitelli, Dario Gregori, Lolita Sasset, Marco Trevenzoli, Vincenzo Scaglione, Sara Lo Menzo, Serena Marinello, Daniele Mengato, Francesca Venturini, Ivo Tiberio, Paolo Navalesi, Annamaria Cattelan

**Affiliations:** 1 Infectious and Tropical Diseases Unit, Padua University Hospital, 35128 Padua, Italy; 2 Unit of Biostatistics, Epidemiology and Public Health, DCTVPH, University of Padova, 35128 Padua, Italy; 3 Hospital Pharmacy Unit, Padua University Hospital, 35128 Padua, Italy; 4 Anesthesiology and Intensive Care Unit, Padua University Hospital, 35128 Padua, Italy; 5 Department of Medicine (DIMED), Padua University Hospital, 35121 Padua, Italy

**Keywords:** cefiderocol, colistin, *Acinetobacter baumannii*, CR-Ab, multi-drug resistant, pneumonia, bloodstream infections

## Abstract

Background. A large increase in multi-drug-resistant *Acinetobacter baumannii*, especially carbapenem-resistant strains, occurred during the first two years of the COVID-19 pandemic, posing important challenges in its treatment. Cefiderocol appeared to be a good option for the treatment of Carbapenem-resistant *Acinetobacter baumannii* (CR-Ab), but to date, the guidelines and evidence available are conflicting. Methods. We retrospectively included a group of patients with CR-Ab infections (treated with colistin- or cefiderocol-based regimens) at Padua University Hospital (August 2020–July 2022) and assessed predictors of 30-day mortality, and differences in microbiological and clinical treatment. To evaluate the difference in outcomes, accounting for the imbalance in antibiotic treatment allocation, a propensity score weighting (PSW) approach was adopted. Results. We included 111 patients, 68% males, with a median age of 69 years (IQR: 59–78). The median duration of antibiotic treatment was 13 days (IQR:11–16). In total, 60 (54.1%) and 51 (45.9%) patients received cefiderocol- and colistin-based therapy, respectively. Notably, 53 (47.7%) patients had bloodstream infections, while 58 (52.3%) had pneumonia. Colistin was combined in 96.1%, 80.4%, and 5.8% of cases with tigecycline, meropenem, and fosfomycin, respectively. Cefiderocol was combined in 13.3%, 30%, and 18.3% of cases with fosfomycin, tigecycline, and meropenem, respectively. At the baseline, the two treatment groups significantly differed in age (patients treated with colistin were significantly older), the prevalence of diabetes and obesity (more frequent in the group treated with colistin), length of stay (longer in the group receiving cefiderocol), and type of infection (BSI were more frequent in the group receiving cefiderocol). The proportion of patients who developed acute kidney injury was significantly higher in the colistin group. By using PSW, no statistically significant differences emerged for mortality or clinical and microbiological cure between the two groups. No independent predictors were detected for hospital mortality or clinical cure, while for the length of stay, the only selected predictor was age, with a non-linear effect (*p*-value 0.025 for non-linearity) on the prolongation of hospital stay of 0.25 days (95% CI 0.10–0.39) at increasing ages (calculated over the IQR). Conclusions. Cefiderocol treatment did not differ in terms of main outcomes and safety profile from colistin-based regimens. More prospective studies with a larger number of patients are required to confirm our results.

## 1. Introduction

The coronavirus disease 19 (COVID-19) pandemic has had a negative impact on antimicrobial resistance, leading to an increase in the spread of multi-drug-resistant (MDR) microorganisms in hospital settings [1,2]. This is mainly to three factors. The first one relies on the measures of contact isolation in COVID-19 areas that were frequently unattended by healthcare providers, due to resource and space constraints [3]. The second factor is the increased risk of COVID-19 patients both to undergo invasive procedures such as mechanical intubation and prolonged hospitalization, with a higher probability of being colonized and infected by MDR agents [4,5]. Lastly, the extensive use of antibiotics to cover possible overlapping bacterial infections increased further the risk of antimicrobial resistance emergence [6,7,8].

In recent years, many warnings have been released by the scientific community and the WHO on the spread of bacteria belonging to the ESKAPE group [9,10].

Among these, carbapenem-resistant *Acinetobacter baumannii* (CR-Ab) species represents one of the main Gram-negative bacteria related to antimicrobial resistance [11]. Its multiple mechanisms of resistance, such as the modification of its outer membrane, efflux pumps, resistance acquisition, and the formation of biofilms, are responsible for the difficulty in the treatment of infections and life-threatening conditions [12].

In 2017, the World Health Organization (WHO, Geneva, Switzerland) added this bacterial species to the list of bacteria for which new antibiotics are urgently needed and with a critical priority level [9]. Indeed, CR-Ab infections are associated with high morbidity and mortality rates [13].

On the one hand, this is often related to the severity of the underlying diseases that characterized hospitalized patients, but on the other hand, it should be mentioned that the therapeutic options available for the treatment of CR-Ab infections are very limited, thus increasing the risk of inappropriate empirical therapy and consequent mortality [13,14].

Some of the most significant predictors of mortality due to CR-Ab infections include prolonged hospitalization, advanced age, male gender, multiple comorbidities, having an infection at the moment of admission, and being admitted to an intensive care unit [15].

The use of advanced life support devices such as continuous renal replacement therapy (CRRT) or extracorporeal membrane oxygenation (ECMO) or the patient’s critical clinical conditions may have a negative impact on achieving the pharmacokinetic/pharmacodynamic (PK/PD) targets required for the treatment of serious infections [16].

Currently, there is no consensus on the optimal treatment schedule or options for CR-Ab infections, and different guidelines report different recommendations and advice [17,18].

To date, colistin (usually combined with other molecules such as meropenem, sulbactam, tigecycline, or fosfomycin) has provided the backbone for the treatment of severe CR-Ab infections, despite its remarkable nephrotoxicity and its poor pharmacokinetic profile, especially in some districts such as the lung [19].

Cefiderocol is a new siderophore cephalosporin, for which in vitro activity against multi-drug-resistant (MDR) Gram-negative bacteria has been reported [20].

Clinical results on its efficacy in treating severe CR-Ab infections are limited and conflicting [21,22,23,24,25,26]. While the “CREDIBLE-CR” trial reported higher rates of mortality at 14 and 28 days in patients with severe CR-Ab infections treated with cefiderocol versus the “best available therapy” [21], many observational data and several case reports highlighted the possible benefits of cefiderocol use on mortality rates and microbiological cure [22,23,24,25,26]. However, since no large and homogenous, randomized clinical trial results are available, additional clinical data on the use of cefiderocol in severe CR-Ab infections are urgently needed to definitively assess its efficacy and reach a consensus among the different guidelines.

In this work, our main objective was to describe our experience with the use of cefiderocol, by assessing any differences in terms of major clinical outcomes (30-day mortality, clinical cure, and microbiological cure) between patients who received colistin- or cefiderocol-based antibiotic treatment for (CR-Ab) infections. As a secondary objective, we aimed to assess the clinical and laboratory predictors significantly associated with the study outcomes.

## 2. Materials and Methods

This is a retrospective cohort study conducted at the Infectious and Tropical Diseases Unit of Padua University Hospital (Padua, Italy). The study was conducted according to the principles of good clinical practice and the Declaration of Helsinki. Patient consent was waived as per Italian law (Italian Drug Agency note, 20 March 2008, Gazzetta Ufficiale Serie Generale no. 76 31/3/2008). Approval was obtained from the ethics committee (5487/2022).

We included all adult hospitalized patients with documented infection by carbapenem-resistant *Acinetobacter baumanii* who received cefiderocol-based antibiotic treatment from August 2021 (the date of its availability at our center) to July 2022. For the control group, we included all patients with documented CR-Ab infections who were treated during the previous year (in which cefiderocol was not available), hence receiving colistin-based antibiotic regimens (August 2020–July 2021). Patients who received colistin-based regimens, in case of pneumonia, also received aerosol colistin administration.

During the whole study period, patients were managed by the same medical staff.

We recorded the presence of concomitant infections, such as those given by fungi, Gram-positive agents, or COVID-19. Patients treated concomitantly with cefiderocol and colistin and with a concomitant Gram-negative agent were excluded.

We classified the type of infection accordingly to CDC criteria in bloodstream infections (BSIs), ventilator-associated pneumonia (VAP), intrabdominal infections (IAIs), urinary tract infections (UTIs), etc. [27,28]. For patients who were diagnosed with pneumonia in the medical ward, we considered only patients who presented the following criteria: the presence of pneumonia documented with chest X-rays, the increased number of inflammatory biomarkers (C-reactive protein (CRP)), and the isolation of *Acinetobacter baumannii* from bronchial aspirate documented on chest X-rays. We recorded the demographic, clinical, and laboratory parameters from medical health records. We also recorded the length of stay; ward of admission (medical, surgical, and intensive care); number and type of comorbidities (cardiovascular, diabetes, obesity, respiratory, renal, psychiatric, and malignancy); SOFA score; APACHE score; white blood count; CRP; procalcitonin; and procedure such as continuous renal replacement therapy (CRRT), continuous venovenous hemofiltration (CVVH), extracorporeal membrane oxygenation (ECMO), and mechanical ventilation. Acute kidney injury was defined according to the Kidney Disease: Improving Global Outcomes (KDIGO) guidelines [29]. All the antibiotic combinations used in either colistin-based or cefiderocol-based antibiotic regimens were recorded. Lastly, we recorded the length of antibiotic treatment. We need to mention that during the first part of the study period, we had a shortage of ampicillin/sulbactam. For this reason, sulbactam is not included among the antibiotic combinations used against cefiderocol.

MICs were classified according to the breakpoints established by the European Committee on Antimicrobial Susceptibility Testing [30,31]. Susceptibility to colistin was determined via broth dilution. For cefiderocol, we used the disk diffusion method (30 mg cefiderocol disks, Liofilchem^®^) to test the growth inhibition zone, as already described by other authors [23,32]. The EUCAST pharmacokinetic/pharmacodynamic (PK/PD) breakpoint MIC values of 2 mg/L were used for *Acinetobacter baumannii* if the inhibition zone diameter for the cefiderocol disk was <17 mm [23,32]. This susceptibility testing was performed only once for the first isolate responsible for the infection per patient. Sensitivity to other antibiotics used in combination, such as meropenem and tigecycline, was assessed using the Vitek^®^ system. Fosfomycin, whenever used, was empirically added and with no sensitivity testing performed, since the agar dilution method were not available at our center.

The primary outcomes of the study were clinical cure (the resolution of signs and symptoms of infection after 14 days from the start of antibiotic treatment) and 30-day all-cause mortality. Secondary outcomes were microbiological cure defined as no further isolation of *Acinetobacter baumannii* from the repeated cultures obtained from the site of the primary infection after 7–14 days from the start of the antimicrobial treatment and the assessment of the clinical and laboratory predictors significantly associated with the study outcomes.

We lastly assessed whether there were any differences in the major clinical outcomes between patients who were treated with cefiderocol as monotherapy versus those treated with cefiderocol combined with other agents.

### Statistical Analysis

Continuous variables were reported as median and quartiles (interquartile range (IQR)). Between treatment groups, comparisons were performed, for continuous variables via the Mann–Whitney test. Categorical data were expressed as frequency distributions, and the Fisher exact test was used to determine if differences existed between groups.

To account for unbalance in treatment allocation (cefiderocol versus colistin), a propensity score weighting (PSW) model (1) was developed using all the available information at baseline (sex, age, and comorbidities) and their interactions up to the fourth degree. Weights were estimated using an energy balance approach (2) and then stabilized by multiplying each unit’s weight by the proportion of units in their treatment group. The estimation of the average treatment effect (ATE) for cefiderocol versus colistin was estimated via generalized linear models (GLMs) (3) with the appropriate link function (binomial logit for in-hospital mortality and clinical cure and gamma log for the length of stay), weighted by propensity scores and adjusted for covariates eventually emerging as unbalanced after PSW estimation. Evaluation of the role of predictors with respect to the three outcomes was performed by estimating unweighted GLMs with the appropriate choice-of-link functions. Covariates were selected in a forward approach and using a Bayesian Information Criterion (BIC) (4). All analyses were performed by using the R System (5) and the WeightIt library (6).

## 3. Results

Over the study period, 116 patients presented a CR-Ab infection (either pneumonia or bloodstream infections). Five patients were excluded since they died before starting any of the studied regimens. Seven patients were excluded because they received both cefiderocol and colistin and presented a concomitant Gram-negative agent (extensively drug-resistant *Pseudomonas aeruginosa*). Therefore, 111 patients were considered for this analysis. The full baseline demographic and clinical characteristics are provided in Table 1. The median age was 69 years (IQR: 59–78), and 75/111 (68%) patients were male.

Overall, 60 (54.1%) and 51 (45.9%) patients received cefiderocol-based and colistin-based antibiotic therapy for the treatment of a documented *Acinetobacter baumannii infection*, respectively. All the isolates of *Acinetobacter baumannii* were sensitive to colistin with a MIC < 0.5 mg/L. In the group of patients who received cefiderocol, all the isolates were susceptible, by presenting a MIC < 2 mg/L.

In patients treated with cefiderocol, 30 (50%) received monotherapy, while the remaining 30 (50%) received fosfomycin, tigecycline, and meropenem in 13.3%, 30%, and 18.3% of the cases, respectively (Table 2). Among the 30 patients who received combined cefiderocol treatment, 8 (26.7%) received meropenem and tigecycline, and 1 received meropenem and fosfomycin (3.3%).

Colistin was combined with tigecycline, meropenem, and fosfomycin in 96.1%, 80.4%, and 5.8% of the cases, respectively. As for the type of infection, 53 (47.7%) had bloodstream infections, while 58 (52.3%) had pneumonia. In the group of patients with pneumonia, 38/58 (65.5%) patients were treated in surgical or medical wards, and 20/58 (34.5%) patients were admitted to ICU and had VAP. Among these, 7/58 (12.1%) patients developed bloodstream infections. The two-treatment group (cefiderocol versus colistin) significantly differed for the following characteristics: age (patients treated with colistin were significantly older); the prevalence of diabetes and obesity (which were more frequent in the group treated with colistin); length of stay (significantly longer in the group receiving cefiderocol-based regimen); and the type of infection (BSIs were more frequent in the group receiving cefiderocol, while pneumonia was more frequent in the group receiving colistin-based treatment). The median duration of antibiotic treatment was 13 days (11–16). Overall, the mortality rate was 43% (48/111), with a higher proportion of death (even if not statistically significant) in patients treated with cefiderocol versus those receiving colistin-based regimens (26/60 (51%) versus 22/51 (37%), *p* = 0.130). Moreover, between the two groups, we did not observe any statistically significant differences in terms of clinical cure and microbiological cure. No adverse reactions or remarkable changes in laboratory findings were observed in the group receiving cefiderocol. The median baseline creatinine level was significantly higher in patients receiving the colistin-based regimen, even though within the normal range. During the antibiotic treatment, 19 patients (17.1%) developed acute kidney injury, with a statistically significant difference (*p* = 0.031) between the group receiving colistin (13/51, 25.5%) and those receiving cefiderocol (6/60, 10%). In both groups, drug dosage was adjusted according to the evolution of renal function.

Of the 36 patients with an ongoing COVID-19 infection, 13 patients died (27.7%). We did not observe any differences in terms of mortality between the two antibiotic treatment groups (7/13 (53.7%) patients treated with cefiderocol-based regimens versus 6/13 (46.3%) patients treated with colistin-based regimens).

Considering cefiderocol monotherapy versus cefiderocol combination therapy, we did not observe any significant difference in the study outcome (Table 3).

By using PSW, no statistically significant differences emerged in any of the three outcomes considered between the group treated with colistin-based regimens versus that receiving cefiderocol-based regimens. No independent predictors of mortality or clinical cure were found. For the length of stay, the only detected predictor was age, with a non-linear effect (*p*-value 0.025 for non-linearity) on the prolongation of the in-hospital stay of 0.25 days (95% CI 0.10–0.39) at increasing ages (calculated over the IQR) (Table 4). In the subgroup of patients with pneumonia, cefiderocol versus colistin treatment showed no differences in any of the study outcomes, while in patients with BSIs, we detected a significant risk reduction in mortality for patients treated with cefiderocol (OR 0.121, 95% CI 0.025–0.578, *p*-value 0.0109).

## 4. Discussion

This study reports the clinical and microbiological outcomes of patients with documented infection by CR-Ab treated with either colistin- or cefiderocol-based antibiotic regimens in the real-life setting of a large third-level university hospital during two years of the COVID-19 pandemic.

To date, only a few active antibiotics are fully active and available for AB-CR infections, and cefiderocol became an important therapeutic option. Recently, IDSA guidelines indicated cefiderocol as a part of combination antibiotic regimens in CR-Ab infections refractory to other antibiotics or in case of intolerance to other agents [18]. A conditional recommendation against cefiderocol for the treatment of infections caused by CR-Ab has been conversely stated by the ECCMID, in which the use of ampicillin/sulbactam, polymyxin, or high-dose tigecycline, or a carbapenem combination therapy has been suggested in sulbactam-susceptible CR-Ab HAP/VAP, in sulbactam-resistant strains, or in cases with a meropenem MIC of <8 mg/L, respectively [17].

Therefore, the resulting therapeutic approach to CR-Ab infections is difficult, and a gold-standard therapy is still lacking. In addition, the mortality rate of CR-Ab infection is high, ranging from 45% to 70% [21,23,25,33,34]. In our cohort, the overall 30-day mortality rate was 43%, with a higher mortality rate observed in patients treated with cefiderocol-based regimens compared with those treated with colistin-based regimens, even though a statistically significant difference was not achieved (37% versus 51%; *p* = 0.31)). Even by performing the propensity-score-adjusted analysis, no benefits were observed with cefiderocol-based treatment. In addition, the microbiological cure rate was comparable between the two groups of therapy (73% versus 67% in cefiderocol- and colistin-treated groups, respectively). Our findings are in line with the results of the CREDIBLE-RCT trial, in which the 28-day mortality rates were 49% in the cefiderocol-treated subgroup versus 18% in the group using the best available therapy, and no advantage to cefiderocol with respect to clinical or microbiological eradication was observed [21].

Our data showed that cefiderocol, even in combination with other antimicrobial agents, did not significantly improve the outcomes of patients with CR-Ab infections when compared with colistin-based regimens. Additionally, in the cefiderocol-treated group, by considering monotherapy versus combination therapy, our results showed that combining cefiderocol with other agents did not improve the major clinical outcomes. This may suggest that cefiderocol should be used as monotherapy. However, many authors have suggested that combination therapy is the best strategy to approach and treat MDR Gram-negative infections [35]. In particular, some previously reported data confirm the efficacy of cefiderocol treatment in combination with fosfomycin, tigecycline, and colistin as rescue therapy for severe CR-Ab infections, even if with more uncertainty for Acinetobacter baumannii infections [36,37,38]. The possible use of sulbactam to treat *Acinetobacter baumanii* infections is also well known [17,39,40]. However, our experience was importantly limited by a temporary shortage of ampicillin/sulbactam combination at our center (where sulbactam is only available in combination with ampicillin) during the first part of the study observation. When using cefiderocol, the role of combination therapy (and which agent would be better used in combination) versus monotherapy remains to be clarified.

However, in severely ill patients with underlying comorbidities or coinfections, it is challenging to determine if poor clinical outcomes are attributable to suboptimal antibiotic therapy or underlying host factors or coinfections.

Regarding mortality in patients with COVID-19, we did not observe any differences between the two treatment groups.

It is well known that late-onset pneumonia represents one of the most common clinical manifestations of *Acinetobacter baumannii*; approximately 55% of CR-Ab infections involve the respiratory system [41], and they generally occur in previously colonized patients.

In our study, there were more patients with pneumonia than with BSIs. In agreement with Falcone et al. [23], the subgroup of patients with CR-Ab pneumonia did not show any significant difference in mortality rates when compared to those undergoing colistin-based regimens, unlike what was observed in cases with BSIs, in which we found a significant risk reduction in mortality for patients treated with cefiderocol (*p* = 0.0109). These results underscore the complex clinical management of CR-Ab pneumoniae in which the differentiation of Ab colonization from infection is still challenging, and patients with underlying conditions (i.e., multiple comorbidities, immunosuppression, etc.) may have significantly worsened treatment outcomes, even if antimicrobial agents were appropriately and timely prescribed.

We found, as expected, that acute kidney injury was significantly more common with colistin-containing regimens than with cefiderocol-containing regimens, confirming the considerable drawbacks of colistin therapy, nephrotoxicity, and the complexity of dosage. It is interesting to note that in our study, the mean baseline creatinine levels were higher in patients treated with colistin-based regimens (even though within the normal range) than in those treated with cefiderocol. Considering the fact that physicians are usually reluctant to prescribe a known nephrotoxic antibiotic to patients with reduced kidney function, one explanatory hypothesis for this observation may be that no other choice was available over the first study period to manage such a difficult to treat infection. Taken together, the data from this study suggest that important advances in the treatment of *Acinetobacter baumannii* infections are urgently needed, and cefiderocol may, at least partially, respond to this issue. In fact, the safety profile of cefiderocol was good, with no adverse events. In addition, no evidence of alterations in iron homeostasis variables was observed.

*Acinetobacter baumannii* has become a major concern for the scientific community due to its extraordinary capability to develop resistance to all antimicrobials long enough to be included in the WHO’s list of “priority pathogens” resistant to antibiotics [42]. In our CR-Ab isolates, cefiderocol resistance was not detected or developed during therapy in all the treated patients. However, it is well known that the extensive use of cefiderocol may lead to an increased prevalence of resistant strains, as has already been documented by the detection of several mechanisms reducing the siderophore receptor gene pirA or mutations involving penicillin-binding proteins [43]. In addition, a recent report on an attractive hypothesis on the reduction activity of the drug in the presence of albumin-rich human fluids may raise some concerns regarding the more appropriate use of this antibiotic to treat CR-Ab infections [44].

This study is affected by some limitations, as a result of which the obtained findings may be non-generalizable, such as the retrospective nature of the study, its limited sample size, patients’ heterogeneity in relation to the ward where the infections occurred, or concomitant coinfections. Unfortunately, we used disk diffusion to test susceptibility to cefiderocol, even if suboptimal [45], since we were not able to perform susceptibility testing for cefiderocol by using broth microdilution. However, even if limited, our study may raise some concerns about the clinical and microbiological efficacy of cefiderocol and its supposed high activity in patients with CR-Ab infections.

In conclusion, our results seem to reduce the initial enthusiasm reported in previous studies of CR-Ab infections treated with cefiderocol [22,24,26]. However, our results, considering their limitations, should be cautiously interpreted. Nevertheless, we believe that cefiderocol should be employed thoughtfully. It can be used in those clinical scenarios in which its intrinsic advantages could be maximized, for example, when other alternatives have been demonstrated to be ineffective, for salvage therapy, or when the risk of toxicity due to colistin is unacceptable [46,47]. Further prospective and randomized clinical studies are needed to provide evidence of the high efficacy of this new molecule and whether it should be combined with other agents in CR-Ab infections.

## Figures and Tables

**Table 1:** Demographics and clinical characteristics of the study population, overall and by treatment group.

Characteristics			Overall n = 111	Cefiderocol n = 60	Colistin n = 51	*p*-Value
Age, years, median (IQR)			69 (59–78)	62 (48–75)	72 (64–81)	<0.001
Gender, male, n (%)			75 (68)	38 (63)	37 (73)	0.300
Number of comorbidities, median (IQR)			3 (2–4)	2.4 (1–4)	3 (2–4)	0.067
Coinfections, n (%)	COVID-19		36 (32)	16 (27)	20 (39)	0.160
	Gram-positive infection		22 (19.8)	11 (18.3)	11 (21.6)	0.670
	Candidemia		7 (6.3)	5 (8.3)	2 (3.9)	0.340
Comorbidities, n (%)	Cardiovascular disease		73 (66)	35 (58)	38 (75)	0.073
	Diabetes		27 (24)	10 (17)	17 (33)	0.041
	Obesity		41 (37)	16 (27)	25 (49)	0.015
	Lung disease		30 (27)	20 (33)	10 (20)	0.100
	Chronic kidney disease		18 (16)	12 (22)	5 (10)	0.091
	Psychiatric disorders		43 (39)	20 (33)	23 (45)	0.100
	Malignancy		19 (17)	9 (15)	10 (20)	0.520
Type of infection, n (%)	Bloodstream infection		53 (47.7)	34 (56.6)	19 (37.2)	0.003
	Pneumonia		58 (52.3)	26 (43.4)	32 (62.8)	
Length of stay, days, median (IQR)			45 (24–70)	52 (32–73)	34 (20–72)	0.023
Ward of admission, n (%)	Medical		51 (46)	22 (27)	29 (57)	0.087
	Surgery		25 (23)	17 (28)	8 (16)	
	ICU		35 (32)	21 (25)	14 (27)	
CVVH, n (%)			5 (4.5)	4 (6.6)	1 (1.9)	0.23
ECMO, n (%)			3 (2.7)	3 (5)	0 (0)	0.1
Mechanical ventilation, n (%)			22 (20)	13 (22)	9 (18)	0.6
SOFA score, median (IQR)			2.5 (1–4.2)	3.5 (2–5)	2 (1–4)	0.072
APACHE score, median (IQR)			10 (7–13)	10 (7.8–13.2)	10 (7–13)	0.890
C-reactive protein, mg/dl, median (IQR)			104 (66–160)	97 (67–160)	110 (61–162)	0.880
Procalcitonin, ng/mL, median (IQR)			0.74 (0.16–3)	0.52 (0.13–1.65)	1.1 (0.2–5.1)	0.270
White blood count, median (IQR)			11.9 (7.6–17.6)	11.8 (7.3–17.6)	11.9 (7.8–17.6)	0.830
Creatinine, mmol/L, median (IQR)			69 (44–101)	64 (36–88)	77 (52–118)	0.042
Acute kidney injury, n (%)			19 (17.1)	6 (10)	13 (25.5)	0.031
Study outcomes		Clinical cure, n (%)	78 (70)	44 (73)	34 (67)	0.440
		Microbiological cure, n (%)	47 (42)	26 (43)	21 (41)	0.820
		Deaths, n (%)	48 (43)	26 (51)	22 (37)	0.130

Legend for Table 1: n = number, % = percentage, IQR = interquartile range, ICU = intensive care unit, SOFA = sequential organ failure assessment, APACHE= acute physiological assessment of chronic health evaluation, CVVH = continuous venovenous hemofiltration, ECMO = extracorporeal membrane oxygenation.

**Table 2:** Cefiderocol- and Colistin-associated treatment.

Regimen-Associated Antimicrobial Agents	Cefiderocol n = 60	Colistin n = 51	*p*-Value
Fosfomycin, n (%) *	8 (13.3)	3 (5.8)	0.19
Meropenem, n (%) *	13 (21.7)	41 (80.4)	<0.001
Tigecycline, n (%) *	18 (30)	49 (96.1)	<0.001
Monotherapy	30 (50)	0 (0)	-

Legend for Table 2: n = number, % = percentage. * Cefiderocol and colistin in combination could have been combined with more than one antibiotic (see details in the Results section).

**Table 3:** Differences in main clinical characteristics and study outcomes for patients who received cefiderocol as monotherapy (n = 30) or in combination (37).

Characteristics		Overall n = 60	Cefiderocol Monotherapy n = 30	Cefiderocol Combination Therapy n = 30	*p*-Value
Age, years, median (IQR)		62 (48–75)	63 (57–71)	60.7 (47.3–74.9)	0.47
Gender, male, n (%)		38 (63)	19 (63.3)	19 (63.3)	1
Number of comorbidities, median (IQR)		2.4 (1–4)	2 (1–3.7)	3 (2–3)	0.31
Coinfections, n (%)	COVID-19	16 (27)	6 (18.2)	10 (33.3)	0.77
	Gram-positive infection	11 (18.3)	3 (10)	8 (26.7)	0.09
	Candidemia	5 (8.3)	2 (6.7)	3 (10)	0.64
Type of infection, n (%)	Bloodstream infection	34 (56.6)	19 (63.3)	15 (50)	0.29 0.29
	Pneumonia	26 (43.4)	11 (36.7)	15 (50)	
Length of stay, days, median (IQR)		52 (32–73)	51 (30–69)	51.5 (33.2–78.5)	0.29
Ward of admission, n (%)	Medical	22 (27)	11 (36.7)	11 (36.7)	1 0.77 0.78
	Surgery	17 (28)	9 (30)	8 (26.7)	
	ICU	21 (25)	10 (33.3)	11 (36.7)	
CVVH, n (%)		4 (6.6)	2 (6.7)	2 (6.7)	1
ECMO, n (%)		3 (5)	1 (3.3)	2 (6.7)	0.55
Study outcomes	Clinical cure, n (%)	44 (73)	23 (76.7)	21 (70)	0.55
	Microbiological cure, n (%)	26 (43)	15 (50)	11 (36.7)	0.29
	Deaths, n (%)	26 (51)	10 (33.3)	16 (53.3)	0.81

Legend for Table 3: n = number, % = percentage, and IQR = interquartile range.

**Table 4:** Propensity-score-adjusted estimates of average treatment effect (ATE) of cefiderocol versus colistin.

	Effect	95% CI		*p*-Value
Mortality	OR			
ATE (Cefiderocol versus. Colistin)	0.810	0.329	1.995	0.647
Age	0.951	0.917	0.986	0.007
Length of stay	Gamma Coefficient			
ATE (Cefiderocol versus Colistin)	8.467	−7.848	24.782	0.315
Age	−0.267	−0.665	0.125	0.180
Clinical cure	OR			
ATE (Cefiderocol versus Colistin)	1.134	0.965	1.333	0.128
Age	0.993	0.986	0.999	0.020

Legend for Table 4: Colistin vs. cefiderocol with regard to mortality, length of stay (LoS), and clinical cure (CC) are further adjusted by age. OR = odds ratio; gamma coefficient is the marginal effect of the gamma-GLM parameter for the LoS (expressed as number of days). ATE effects are adjusted by age.

## Data Availability

Data are available on reasonable request from the corresponding author.
